# The role of miRNAs in the development of Super-Tregs as a potential therapy for neurodegenerative diseases

**DOI:** 10.3389/fimmu.2026.1708031

**Published:** 2026-02-04

**Authors:** Kamalika Mukherjee, Suvendra N. Bhattacharyya

**Affiliations:** 1 Department of Anesthesiology, University of Nebraska Medical Center, Omaha, NE, United States; 2 Department of Pharmacology and Experimental Neuroscience, University of Nebraska Medical Center, Omaha, NE, United States

**Keywords:** alzheimer’s disease, amyloid proteins, miRNA, neurodegeneration, neuroinflammation, Parkinson's disease, T regulatory cells

## Abstract

Regulatory T cells, or Tregs, are designed to limit unnecessary inflammation and serve as a safeguard mechanism to prevent tissue damage caused by heightened inflammatory responses from activated macrophages or effector T cells. Impaired Treg function has implications in autoimmunity and neuroinflammation. Neuroinflammation triggered by amyloid proteins and protein aggregates accelerates neurodegeneration due to increased cytokines and chemokines in the brains of individuals with Alzheimer’s Disease and Parkinson’s Disease. A simple approach involves preventing inflammation by suppressing T-effector cell activity in affected brains through boosting Tregs’ function. Super-Tregs, with enhanced anti-inflammatory properties, can be engineered *in vitro* to combat inflammation in various tissues and, after homotropic transfer to the target tissue, prevent damage caused by inflammation. The development of Super-Tregs can be achieved through specific genetic and epigenetic modifications. Efforts to generate Super-Tregs utilizing miRNAs and miRNA-containing extracellular vesicles hold promise in treating neuroinflammation with miRNA-engineered Super-Tregs. In this review, we discuss the potential, progress, challenges, and limitations of Super-Treg development and their application in the treatment of neurodegeneration.

## Introduction

The innate and adaptive immune systems employ protective mechanisms to combat invading pathogens and the accumulation of defective or cancerous cells, thereby maintaining immune homeostasis (1). Both innate and adaptive immunity are essential in combating pathogenic agents across various tissues, including the brain (2). CD4+ T effector (Teff) cells differentiate into Th1, Th2, or Th17 cells, which contribute to inflammatory responses, and any imbalance in their tissue abundance is linked to inflammatory diseases (3). Th1 cells produce pro-inflammatory cytokines like IFN-γ and TNF-α that activate macrophages and cytotoxic T cells to contain intracellular pathogens and promote cell-mediated immunity. In contrast, Antigen-Specific Tregs promote humoral immunity by secreting IL-4 and IL-5, stimulating B cell proliferation and antibody production, enhancing defense against extracellular pathogens and allergens. Th17 cells regulate responses against bacteria and fungi, producing IL-17, IL-22, and IL-23, which recruit neutrophils and phagocytes to clear pathogens and aid tissue repair. The balance among the Th cell types prevents excessive immune activation and autoimmunity. Both intrinsic and extrinsic immune and environmental factors regulate this process (4–7). Teffs are pro-inflammatory, and during microbial infections, they protect the host against infecting agents by promoting immune-mediated microbial pathogen clearance (8–10).

While Teffs promote inflammation and autoimmunity, CD4+ CD25+ FOXP3+ regulatory T cells (Tregs) counteract persistent inflammatory responses (6). Treg cells monitor immune overactivation to prevent damage caused by excessive pro-inflammatory molecule production, thereby promoting anti-inflammatory and suppressive immune responses (5, 11). Tregs produce IL-10 and transforming growth factor beta (TGF-β) to inhibit immune activation (12). Hence, they resolve inflammation and maintain self-tolerance. This suppresses autoreactive Teffs (11, 13). The immune system balances pro-inflammatory (IL-1β, IL-6, TNF-α) and anti-inflammatory (IL-4, IL-10, IL-11, IL-13) cytokines, key regulators of inflammation. Modulating responses is vital for developing new therapies for infectious, inflammatory, and degenerative diseases (14).

The goal of immunoregulation is to produce Tregs with potent anti-inflammatory properties for effective control of inflammation. This review discusses Treg-based therapies, their limitations, and predicts progress toward ‘Super-Tregs” to address overactive immune responses in neurodegenerative diseases like Alzheimer’s and Parkinson’s (AD and PD). The review will discuss how genetic and epigenetic manipulations, particularly involving anti-inflammatory microRNAs (miRNAs) and extracellular vesicles, may facilitate the transition of Tregs into Super-Tregs. This transition can combat degenerative and autoimmune disorders (9–11). Generating extracellular vehicles (EVs) with anti-inflammatory properties is another promising aspect. Recent years have seen the immune regulatory responses mediated by extracellular vesicles (EVs) (15). EVs carry proteins, lipids, RNAs, and DNA fragments from cellular origins (16), play pivotal roles in intercellular crosstalk, and act as carriers of immunoregulatory factors (17, 18). As EVs regulate the host immune response and possess neuroprotective functions, we hypothesized that they might also enhance Treg activity and serve as mediators of Treg activation to produce pro-Super-Tregs, a subset of Tregs with enhanced immune-repressive function but without any genetic-level alteration (19).

## Disease defending Tregs

The “ignited” immune system causes widespread tissue damage in the face of degenerative disease. Tregs combat inflammation, functioning as the body’s natural “brake” on accelerated immune responses that include pro-inflammatory cytokines. Tregs are classified into three populations (20, 21). Thymus-derived Tregs (tTregs), or natural Tregs, differentiate from CD4+ progenitors. Characterized by CD25+ FOXP3– or CD25– FOXP3low expression, they are considered “natural” Tregs and have a high affinity for self-antigens (22). Peripheral-derived Tregs (pTregs) are induced by antigen stimulation of naive T cells. This occurs in the spleen, lymph nodes, and gut-associated lymphoid tissues (23). Induced Tregs (iTregs) develop from naïve CD4+ T cells when stimulated with anti-CD3, IL-2, and TGF-β. Their manipulability offers great potential for cell based therapies.

Although no single marker uniquely identifies iTregs, FOXP3, also known as forkhead box P3 or scurfin, is a key protein necessary for Treg suppressive functions (24). Because Tregs can suppress Teff activation, proliferation, and pro-inflammatory cytokine production (25–27), they are targets for the treatment of autoimmune diseases (25, 27). Tregs also contribute to tissue repair, regeneration, and angiogenesis (25, 28–30). Treg dysfunction contributes to the development of autoimmunity in lupus, multiple sclerosis, and type 1 diabetes (31, 32). Tregs can prevent organ rejection. However, excessive Treg cell activity can preclude antitumor immune responses (33–35) and promote tumor growth (26, 33, 35, 36). Thus, enhanced Treg responses can precipitate diseases (37).

## Tregs and neurodegenerative diseases

### Tregs in AD

Neuroinflammation in AD and PD results from inflammatory environments caused by amyloid beta plaques, phosphorylated Tau, or synuclein oligomers (38). Myeloid and T cell immune responses affect the pathobiology of AD, PD, and amyotrophic lateral sclerosis (ALS) (39–41) through direct, innate, and adaptive immune-mediated cytotoxicity (39, 42–44). Depending on their type and activation state, Teffs and Tregs can either exacerbate or mitigate neuroinflammation (42, 45, 46). CD4+ T cells, specifically Th1, Th17, and gamma-delta T cells, accelerate neurodegeneration by activating brain mononuclear phagocytes (MPs), monocytes, brain macrophages, dendritic cells, and microglia (39, 47–49), whereas Tregs suppress this process (39, 50–52).

Alzheimer’s disease (AD) is a progressive brain disorder that affects memory, cognition, and reasoning (53). AD is the most common cause of dementia in older adults, affecting about 6.9 million in the U.S. aged 65+, mostly over 75, and 55 million worldwide (54). The disease features neural cell death, brain shrinkage, amyloid-β plaques, and tau tangles, hallmark signs of AD (55). Effective treatment to manage the disease’s signs and symptoms and improve quality of life is urgently needed.

AD has genetic and non-genetic causes. Rarely, early-onset Alzheimer’s (EOAD) results from mutations in APP, PSEN1, and PSEN2. More often, late-onset AD (LOAD) occurs after 65, usually sporadic but with genetic risk factors. The primary genetic risk factor for LOAD is the APOE4 gene variant (56). Recent findings indicate that microglial-driven T cell infiltration contributes to neurodegeneration in tauopathies. Additionally, inhibiting interferon-γ and PD-1 signaling significantly reduces brain atrophy (57).

Teffs and Tregs have opposing roles in AD. Teffs exacerbate neuroinflammation, whereas Tregs can be protective early on by enhancing microglial and astrocytic activity to clear amyloid-beta and reduce inflammation (45, 58). However, impaired Treg function can contribute to neurodegeneration in the aging brain (45, 59, 60). Restoring the immunosuppressive environment by amplifying Treg activity could be a disease-combating therapy for neuroinflammation (59). Current therapeutic advances involve Treg cell-based and IL-2-based therapies to boost Treg cell populations, along with innovative approaches to improve their delivery to the affected brain (61, 62).

### Tregs in PD

In the U.S., about 1.1 million have Parkinson’s, expected to reach 1.2 million by 2030, with nearly 90,000 new cases annually. Globally, the WHO estimates over 8.5 million people have PD, with numbers rising (63). PD is a progressive neurodegenerative disorder that primarily affects movement and leads to balance, coordination, and muscle control deficits (64). The disease involves nigrostriatal degeneration, a loss of dopamine-producing neurons that control movement. Symptoms include tremor, rigidity, and bradykinesia (64). Non-motor symptoms include sleep, depression, anxiety, memory, and fatigue difficulties (65).

The exact cause of Parkinson’s disease (PD) is unknown. While genetic factors play a causal role, most cases of PD are sporadic, resulting from complex interactions between genetic and environmental factors. Exposure to pesticides and air pollution may increase the risk of disease (66). 10-20% of PD cases are genetic, with 80-90% sporadic. Age is the main risk, as most develop after 60 (67).

The neuroinflammatory response in the nigrostriatal system is a prevalent pathological characteristic of Parkinson’s Disease (PD). This response is evident in neurotoxicant and α-synuclein models of PD, as well as during dopaminergic neuron degeneration (68). The NLRP3 (NLR Family Pyrin Domain-Containing 3) inflammasome is a primary driver of PD inflammatory neurotoxicity (68). Chronic activation of the NLRP3 inflammasome is initiated by misfolded α-synuclein aggregates that accumulate and propagate throughout disease progression (69). Alpha-synuclein (α-syn) plays a key role in Parkinson’s disease (PD), marked by dopaminergic neuron loss in the substantia nigra pars compacta (SNpc). It accumulates in disorders like PD and dementia with Lewy bodies (DLB), forming Lewy bodies and neurites linked to neuronal degeneration (70). Mutations in the SNCA gene, which encodes α-syn, cause familial PD and increase the risk of sporadic PD. Animal models that replicate dopaminergic neuronal loss and α-syn aggregate formation are used to study disease onset and progression (71).

Like in AD, Tregs play a crucial role in inflammatory response and PD progression (72). Th17 cells promote dopaminergic neuronal death via LFA-1/ICAM-1 interaction in PD models (73). α-syn stimulation reduces FOXP3 expression and increases IL-17A by promoting RORC transcription in Tregs and Th17 cells, boosting Th17 differentiation while decreasing Treg numbers and function (74). Functional deficits in Tregs contribute to dopaminergic neuronal damage. Activated microglia and the release of pro-inflammatory cytokines are significant factors in the pathogenesis of PD (75, 76). Immune imbalance, characterized by ineffective Tregs, may contribute to chronic neuroinflammation and accelerate dopaminergic neuronal loss (74, 76).

**Figure 1:**
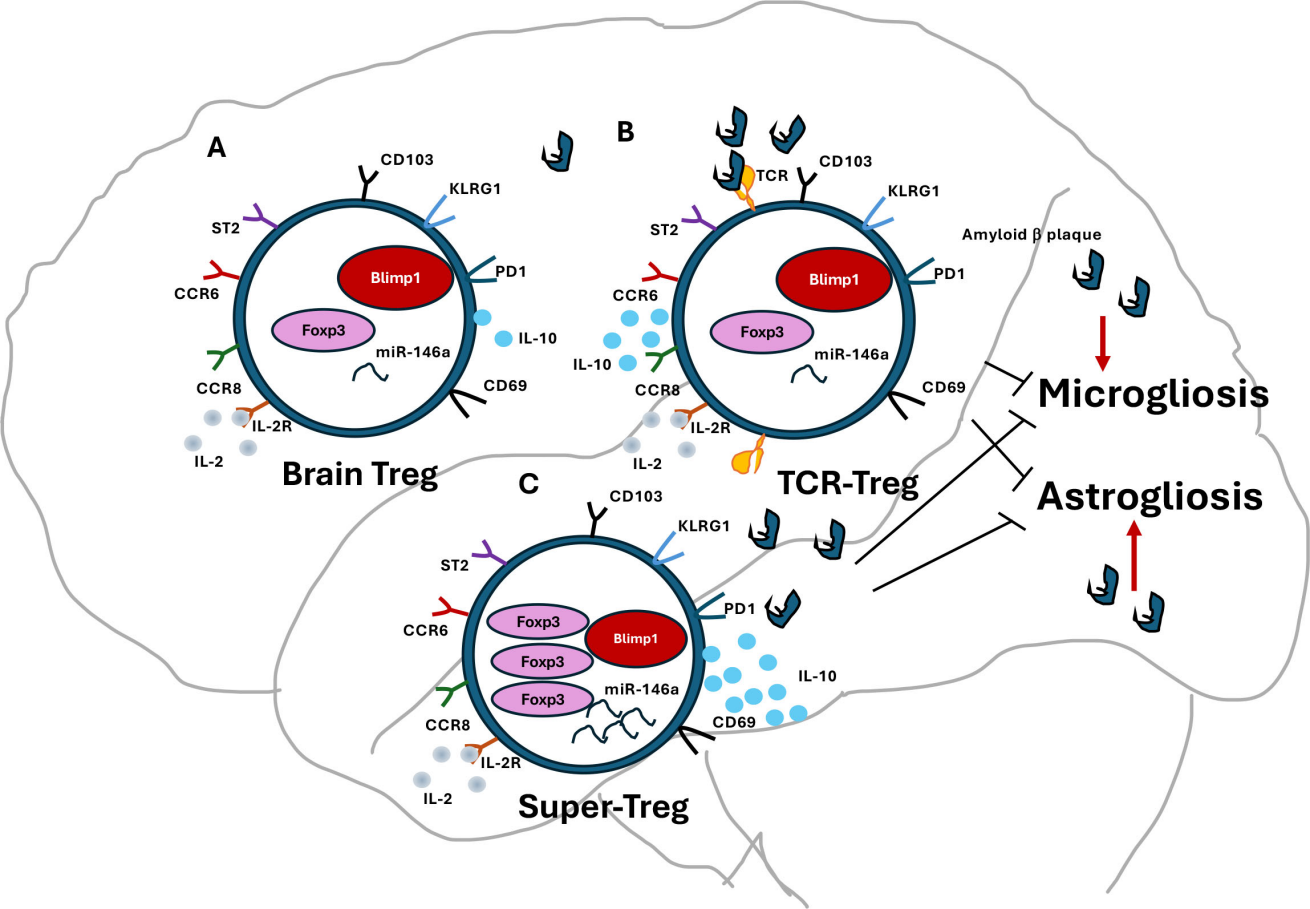
Tregs are in control of neuroinflammation. Brain-resident Tregs are not static; they are dynamic entities that undergo rapid replenishment and exchange with peripheral Tregs. **(a)** In a healthy brain, resting Tregs exhibit low levels of FOXP3 and Blimp1 expression, along with reduced IL-2R and IL-2 levels, indicating a quiescent state. **(b)** The amyloid beta oligomer-specific TCR-Tregs are ex vivo-generated Tregs, derived from induced Tregs (iTregs) through genetic engineering to express TCRs specific to amyloid beta oligomers. Following this modification, they are adoptively transferred into mice, where they become enriched in the brains of APP/PS1 mice, which possess mutations in the APP and PS1 genes. Once inside, these Tregs are activated to express heightened levels of IL-2R and IL-10, playing a crucial role in suppressing activated microglia and astroglia. This mechanism effectively reduces the production of inflammatory cytokines in the brains of individuals with Alzheimer’s disease (AD). **(c)** Super-Tregs can be generated from iTregs that have been manipulated to express elevated levels of FOXP3 and Blimp1, alongside increased expression of miR-146a and IL-10. These Super-Tregs are expected to demonstrate enhanced homing capacity to the AD brain and exhibit robust immunosuppressive properties.

Thus, regulatory T cells (Tregs) are key in Alzheimer’s and Parkinson’s diseases. Dysfunctional Tregs promote inflammation, leading to neuronal damage. Research explores therapies to boost Treg function, potentially reversing neuroinflammation and improving future treatments (**Figure 1**).

## Treg-based therapies for neurodegeneration

Tregs can induce microglia and astrocytes to engage in phagocytosis and clear Aβ deposits in the early stages of AD (58). Tregs can also help protect neurons and improve cognitive function (58, 77). AD pre-clinical studies demonstrate that antigen-specific TCR-Tregs can reduce Aβ plaque formation, improve learning and memory, and reverse cognitive deficits (78, 79). Treg numbers decline during disease, indicating a loss of regulatory function. Strategies to restore Treg function through expansion are being explored (59, 80–82). Tregs with TCRs designed to target amyloid beta oligomers are highly effective in reversing disease phenotypes and pathology in the APP/PS1 mouse model of Alzheimer’s disease. Transfer of TCR(Aβ)-Tregs expressing an Aβ-specific TCR led to sustained immune suppression, reduced microglial reaction, and lower amyloid load (78). Aβ-specific Tregs improve symptoms in 5xFAD mice, transgenic for mutant human amyloid beta precursor protein (APP) with Swedish, Florida, and London mutations, and human PS1 with M146L and L286V mutations. Transgene expression, driven by Thy1 promoter in neurons, reflects key aspects of Alzheimer’s amyloid pathology. Following Aβ stimulation, isolated and expanded CD4+CD25+ Tregs recover cognitive function when adoptively transferred into 5xFAD mice (79).

In PD, decreased Treg cell numbers and impaired suppressor function have been reported (83). Research indicates that Tregs taken from the blood of Parkinson’s Disease (PD) patients show diminished immunosuppressive abilities and functional weaknesses when compared to Tregs sourced from healthy individuals (84). PD patient-derived Tregs fail to suppress activated myeloid cells (85). Treg-based therapies restore immune balance by expanding or modulating Treg cells through adoptive cell therapy (*ex vivo*) and *in vivo* strategies such as low-dose IL-2 therapy. The aim is to increase Treg numbers or enhance their suppression of effector T cells to reduce inflammation and restore immune tolerance (60, 72, 78, 79, 86). Recently, the regulatory T cell-selective IL-2 receptor agonist rezpegaldesleukin (REZPEG) has been a promising therapeutic agent in development (87). Treg expansion by CD28 superagonistic antibodies attenuates neurodegeneration in A53T-α-synuclein PD mice (88). Expanding Tregs *ex vivo* and transferring them to mice restored their suppressive function and protected dopaminergic neurons (72). Co-transplantation of Treg cells and stem cell-derived dopaminergic neurons safeguards grafted cells from damage while enhancing therapeutic outcomes in rodent models of PD (89). TNF-α antibodies and CD4+CD25+ Treg cell transfer reduced Th1 cells while increasing Treg cells in the brains of experimental PD mice (90).

α-synuclein can also impact Treg function. Studies suggest that α-synuclein promotes Th17 cell differentiation, thereby driving neuroinflammation and impairing Treg function (74). One study showed α-synuclein promotes Th17 differentiation and impairs Treg function. Another found that α-synuclein-specific Tregs, generated by dendritic cells presenting α-synuclein, had neuroprotective effects in PD models (72, 74, 91).

A significant challenge with Treg-based therapy is achieving specificity. Engineered T cells expressing chimeric antigen receptors (CARs) provide enhanced cell-based responses by enabling T cells to recognize and eliminate tumor cells independently of the major histocompatibility complex (MHC) (92, 93). CARs are synthetic receptors designed to recognize tumor antigens, empowering T cells to target and destroy tumors. Developing antigen-specific Tregs shows promise in autoimmune diseases and Type 1 diabetes. Insulin-specific chimeric antigen receptor-modified Tregs may provide a new immunotherapy for Type 1 diabetes (94). Systemic Lupus Erythematosus (SLE) is a progressive condition marked by immune tissue damage and lymphoid changes. A therapy using Tregs overexpressing FOXP3 and an anti-CD19 CAR (FOXP3CAR-Tregs) can effectively inhibit B-cell proliferation and activity, which are key to SLE development (95).

Such approaches also enable Treg cells to recognize and bind to specific antigens associated with PD, such as alpha-synuclein (57, 72, 96). PD is characterized by neuroinflammation, in which immune cells in the brain are activated and contribute to neuronal damage (88, 97). Immune-based manipulations can help modulate this inflammatory response by suppressing the activity of pro-inflammatory immune cells (46, 89). By mitigating inflammation, Tregs may help slow the progression of neuronal loss, a hallmark of PD (72, 97). In preclinical studies, antigen-specific Tregs have shown promise in improving motor function in mouse models of Parkinson’s disease (72, 97). Such approaches can shift microglial polarization toward a more anti-inflammatory state (72, 98), thereby preventing neuroinflammation in PD. These Treg modifications for PD are still in the early stages of development. Further studies are needed to explore the optimal design, delivery methods, and long-term effects (72, 99). Cells are now being engineered to react against beta-amyloid plaques (100). By targeting these amyloid or α-synuclein, CAR-Tregs can clear plaques, reduce neuroinflammation, and promote neuroprotection (58). The ability to engineer Tregs to recognize specific antigens enables researchers to identify a more precise and targeted therapeutic approach (58, 100, 101).

## Super Tregs in neurodegeneration

**Figure 2:**
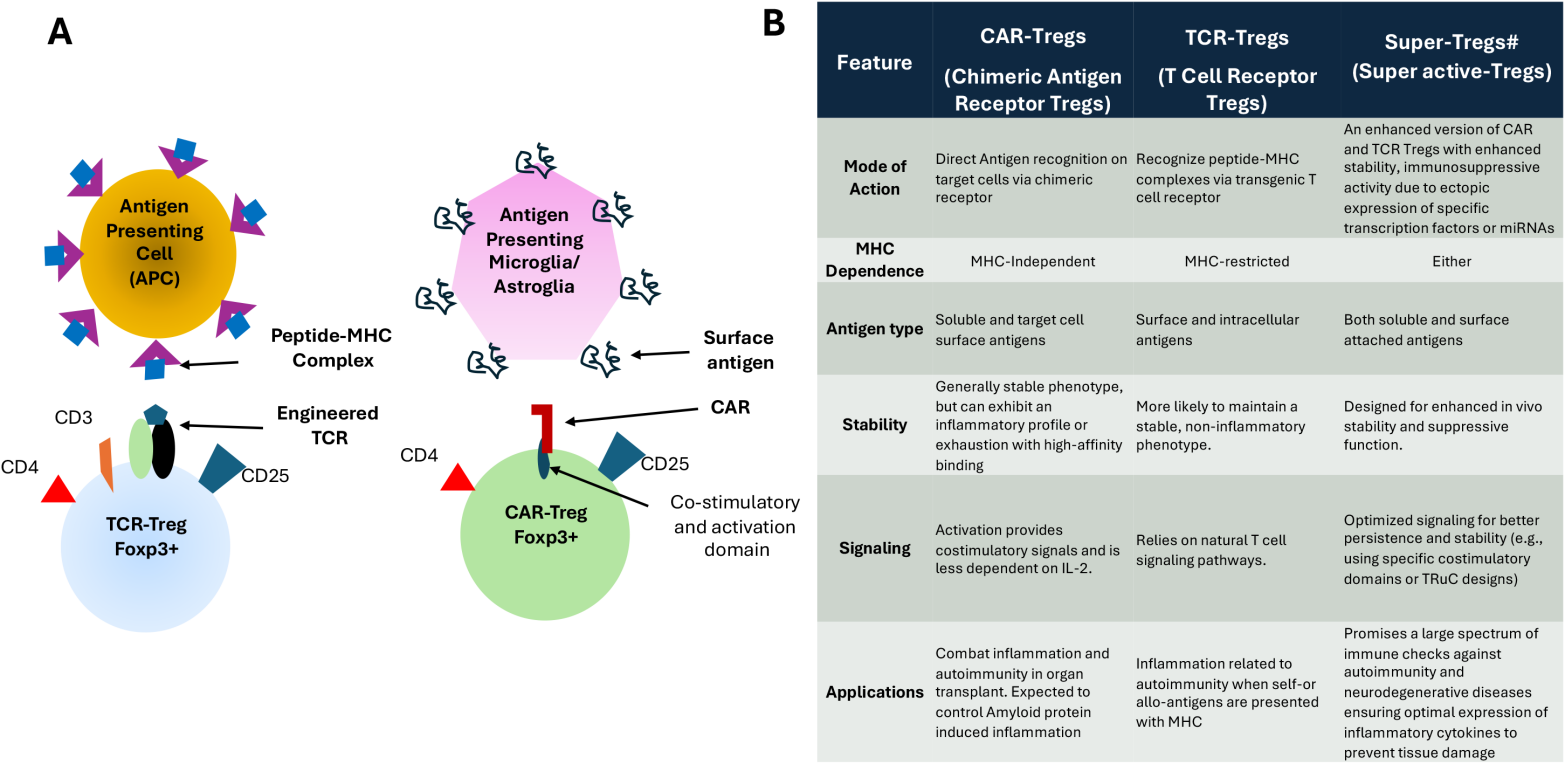
Differences between CAR-Tregs and TCR/Antigen Specific-Tregs in action and their potential conversion to Super-Tregs. **(A)** The Diagrams showing the mode of antigen recognition difference between CAR-Tregs and TCR-Tregs. **(B)** Summary of the difference betweens TCR-Trgs and CAR-Tregs and possible relation to Super-Tregs that can be generated from either CAR-Tregs or TCR-Tregs by manipulation of target genes. Super-Tregs are engineered Tregs with “superpowers” such as enhanced stability, migration, and potency, achieved via genetic edits (e.g., CRISPR), designed to overcome the limitations of first-generation CAR-Tregs and TCR-Tregs, including instability and off-target effects.

Why do we need Super Tregs? Tissue-resident Tregs can suppress inflammation, but their balance with Teffs shifts as amyloid or Lewy body deposits build up in the brain. This results in fewer Tregs that inhibit inflammation. Managing inflammation is challenging, especially in increasing Treg numbers and function in the diseased brain. Super-Tregs may help by boosting the proportion of antigen-specific Tregs in AD and PD brains. However, their effectiveness drops in severe inflammation caused by Teffs in these diseases. Thus, more resilient Tregs capable of counteracting Teffs and maintaining immunosuppression are needed. “Super-Tregs,” with enhanced immunosuppressive abilities, are engineered or modified Treg cells that express key transcription factors or epigenetic regulators at high levels. They hold promise for neurodegenerative diseases like AD and PD by reducing inflammation and supporting neuroprotection. These cells can be created and modified to reduce harmful immune responses and boost the brain’s natural healing processes. Super Tregs are generated from a patient’s T cells that are engineered to exhibit specific traits, enabling them to suppress inflammation and support neuroprotection (19, 102). Boosting Treg activity has shown promising results for various neurodegenerative diseases (45, 84, 102, 103). Treg-based therapies could offer a novel approach to treating these debilitating conditions by modulating the immune response and promoting neuroprotection. Some research has focused on inducing Tregs to enhance their numbers or functions, while others have investigated the use of engineered Tregs with specific targets (104). For instance, Coya Therapeutics develops therapies that utilize enhanced Tregs and Treg-derived exosomes (19, 102). In summary, “Super-Tregs” offer a promising approach to treating neurodegenerative diseases by harnessing the immune system’s capacity to protect neurons and promote brain health (45) (**Figures 1**, **2****).**

### Super-Tregs generation by genetic manipulation

Super-Tregs, or enhanced function Tregs, can be produced via genetic engineering to boost their effectiveness, longevity, and target specificity for therapeutic use. These alterations aim to develop a more potent Treg cell phenotype that can efficiently suppress aberrant immune responses and may help treat autoimmune diseases or prevent transplant rejection (105, 106). However, Tregs may be functionally impaired or insufficient in a specific disease context. Adoptive Treg therapy, in which Tregs are infused into patients, holds promise but has limitations, including the instability of FOXP3 expression and the potential for impaired suppressive function (107). There is an urgent need for a strategy to boost Treg function to combat inflammation.

### Enhancing Tregs function through genetic engineering

Genetic engineering techniques enable the creation of “Super-Tregs” with enhanced capabilities (105, 106). Modifications can direct Tregs to identify and suppress specific immune responses, potentially improving their therapeutic efficacy (108). Genetic engineering improves the suppressive ability and lifespan of T regulatory cells (Tregs). Advanced genetic techniques now enable the design of customized “designer” Tregs that address the limitations of polyclonal Tregs and introduce new features. These designer Tregs are categorized into three phases: the FOXP3 engineering phase, where the FOXP3 transcription factor promotes the conversion of conventional T cells into Tregs; and the antigen-specificity phase, which employs transgenic T-cell receptors and chimeric antigen receptors to enhance Treg therapy effectiveness (109); and the Super-Treg stage, using advanced genome-editing techniques to refine existing and new engineering approaches to convert regular Tregs into Super-Tregs (110).

The transcription factor Blimp-1 is essential for regulating the FOXP3+RORγt+ Treg subset. Its intrinsic expression in these cells is necessary to inhibit the production of Th17-associated cytokines. Blimp-1 acts as a molecular switch, inhibiting inflammatory activity in FOXP3(+)RORγt(+) regulatory T cells and restraining their immune-activating function by blocking IL-17 production (111). Future strategies may involve generating allogeneic Super-Tregs from donor cells, creating a readily available source for therapy. Interestingly, therapeutically expanded human regulatory T cells are found to be highly suppressive after induction of CD73 expression by HIF1A (112).

Developing methods to eliminate Super-Tregs when necessary is crucial, as it may prevent excessive immune suppression. To that end, a report on a reversible knockout model that re-expresses *Brg1*, a chromatin remodeler, in conjunction with a severe endogenous proinflammatory environment, suggests that it can convert defective Treg cells into powerful, super-activated Treg cells (Super-Tregs) reversibly (113).

### Tools for gene editing and gene transfer to Tregs

Efficient delivery of gene-editing components into the cell nucleus is crucial for successful genetic manipulation (114). Super-Tregs can be engineered to target specific antigens, thereby enhancing their ability to suppress immune responses directed against those antigens. Auto-antigen Smith-specific regulatory T cells impede the progression of lupus nephritis (115). Inducing FOXP3 expression can improve the safety and antigen-specific function of engineered regulatory T cells (116). CTLA-4 is crucial for Treg-mediated suppression of immune cells. It interacts with CD80 and CD86 on antigen-presenting cells, competing with CD28 for these ligands and blocking T-cell activation. Genetic modifications may increase the expression of immunosuppressive molecules like CTLA-4 or FOXP3, leading to more potent suppression of effector T-cell activity (112, 117). Tregs can also be modified to resist apoptosis and survive longer in the body, thereby maintaining their suppressive function (112).

### Genetic modifications convert Tregs to super Tregs

Gene delivery into Tregs uses techniques such as viral vectors to modify their function or regulate gene expression, harnessing Tregs’ ability to promote tolerance and suppress immune responses for therapeutic purposes (118–120). Viral vectors, including retroviruses, lentiviruses, and adeno-associated viruses (AAVs), are commonly used for effective gene delivery in Tregs because they can transduce T cells. These viruses can integrate their genetic material into the host cell’s genome, resulting in stable gene expression. Non-viral methods, such as electroporation and nanoparticles, also offer options for temporarily delivering genes into Tregs, providing advantages for administering genes or gene-editing tools without the risk of viral integration.

**Figure 3:**
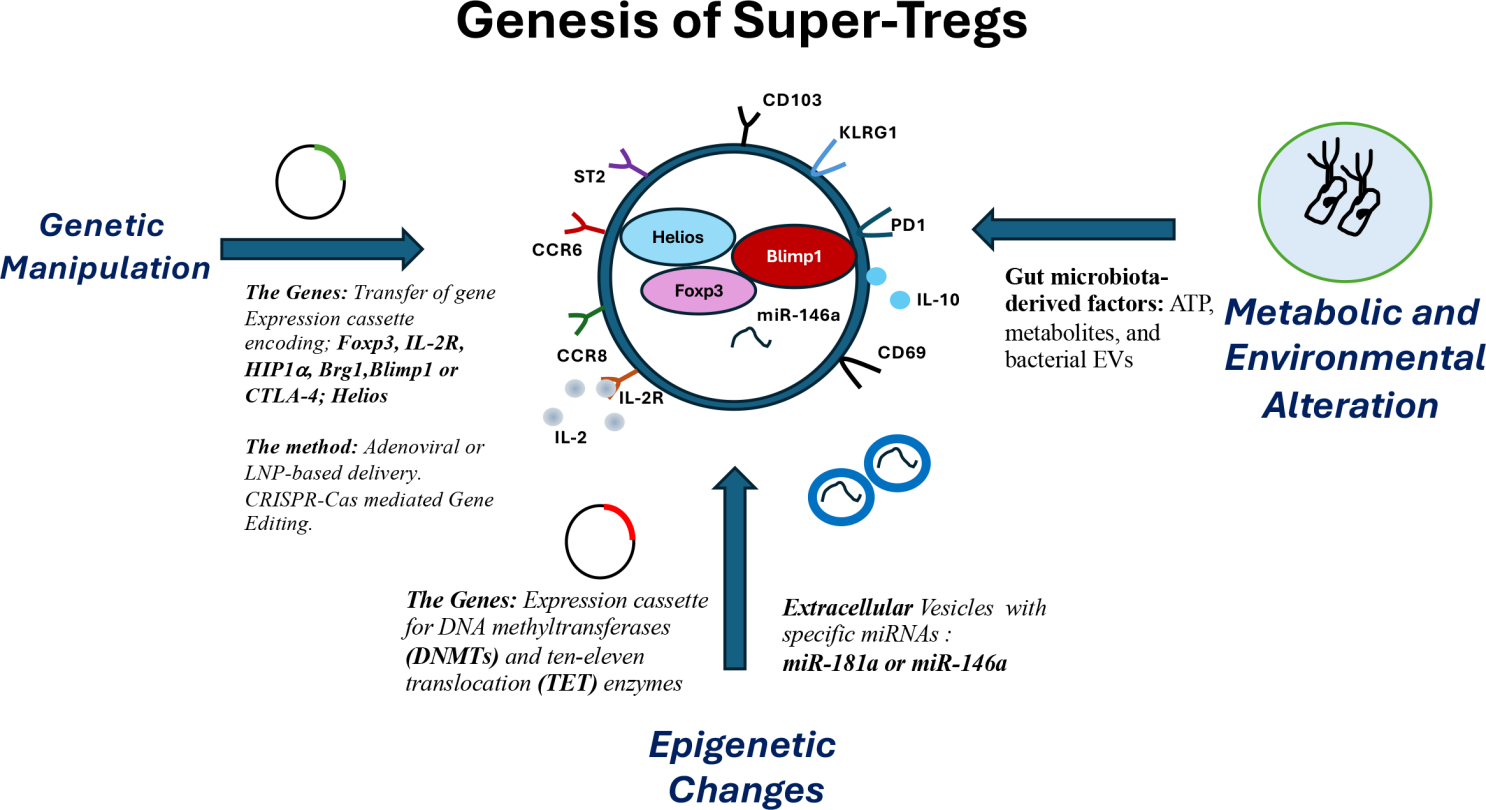
Strategies for the generation of Super-Tregs for therapeutic use. To achieve super Tregs that exhibit high levels of FOXP3 and IL-10 production, various strategies can be employed. One effective method is to transfer gene expression cassettes that introduce genetic and epigenetic modifications, leading to the upregulation of FOXP3 by targeting FOXP3 and its associated pathways. Additionally, the expression of specific miRNAs can be induced to facilitate this conversion. Another promising approach involves utilizing extracellular vesicles from various sources that carry immunosuppressive miRNAs, which can transform standard Tregs into super Tregs. Furthermore, factors derived from gut microbiota and bacterial extracellular vesicles are also recognized as potential modulators in the transition from regular Tregs to super Tregs.

Additionally, transposon technology is another non-viral approach for delivering DNA to Tregs (118). Tregs can be engineered to express specific antigen-specific TCRs or molecules that induce tolerance to those antigens, helping to prevent autoimmune diseases (121). Modified Tregs can be used to suppress immune responses to tumors or enhance the efficacy of cancer immunotherapy (122). They can also serve as delivery vehicles for therapeutic genes, leveraging their ability to migrate to specific tissues and suppress immune responses, thereby enhancing the long-term benefits of gene therapy (121, 123). CRISPR-Cas9 technology allows for accurate genetic editing of regulatory T cells (Tregs), enhancing their function and therapeutic potential for diseases like autoimmune disorders, transplant rejection, and cancer. Using CRISPR for engineering provides a promising approach to develop targeted Treg therapies by modifying genes that influence Treg stability, function, and specificity (124). Advanced immune-based technologies engineer Tregs to target antigens specifically, boosting their ability to suppress immune responses. This aids in treating autoimmune diseases and preventing transplant rejection. Compared with traditional polyclonal Tregs, these methods offer advantages such as stronger immunosuppression, stable function, and improved migration to target sites (100, 125). Maintaining the suppressive function of Tregs is crucial during gene delivery and engineering to ensure effective regulation of immune responses. Specific gene-delivery methods, such as viral vectors, may induce toxicity or elicit immune responses; therefore, it is essential to carefully consider the vector used and its impact on Treg cells (120, 126, 127). Ensuring engineered Tregs target the desired antigen or tissue specifically, without affecting other tissues or cell types, is crucial for their therapeutic efficacy and safety (128). Methods such as Zinc Finger Nucleases (ZFNs) (129) and Transcription Activator-Like Effector Nucleases (TALENs) can induce DNA double-strand breaks (130), enabling targeted modifications to the Treg genome. We discuss these strategies in the next section (**Figure 3****).**

### Epigenetics of super-Tregs generation

Epigenetic strategies aim to regulate Treg cells by modifying DNA methylation and histone acetylation, which boosts their function and stability. Through targeting these epigenetic modifications, scientists strive to develop “Super-Tregs.” These changes influence gene expression without changing the DNA sequence itself. In the case of Tregs, such modifications are vital for preserving their identity and function (131, 132). Current research investigates Treg-specific epigenetic mechanisms, like super-enhancers. Tissue Tregs have unique transcriptomes due to overlapping tissue-restricted open chromatin regions on top of those accessible in the spleen, identified by super-enhancers and specific histone modifications. Moreover, accessible chromatin in Tregs from non-lymphoid tissues frequently includes binding motifs for a few transcription factor (TF) families (133). These TF motifs, especially GATA and bZIP, are in core tissue Treg regions and are vital for maintaining gene programs for Treg function across tissues (133).

By investigating epigenetic signatures, we can engineer Tregs that are more stable and effective. Treg-specific super-enhancers (Treg-SEs) are activated during the early stages of Treg development, before the expression of the crucial Treg marker FOXP3. These super-enhancers are linked to Treg-specific gene expression and play a role in maintaining the epigenetic landscape of Tregs (134, 135). Recent research suggests that variations in regulatory T cell-specific epigenomic regions influence susceptibility to autoimmune diseases (136). A report shows that Treg-specific CpG hypomethylation corresponds with Treg cell super-enhancers that dictate their development. Notably, FOXP3 operates in a multimodal manner to directly promote or inhibit transcription in response to context and interacting partners, thereby influencing Treg cell characteristics. This suggests that altering specific co-factors may shift FOXP3’s function from repressive to activating, ultimately affecting Treg’s fate (137) (**Figures 2**, **3****;****Table 1****).**

**Table 1:** Potential gene targets for conversion of Tregs to super Tregs.

Name of the gene	Target pathway in Tregs	Possible effect on Tregs	Reference
FOXP3	DNA methylation, histone modifications, and post-translational modifications, governs the development and optimal suppressive function of Tregs.	Governs the development and optimal suppressive function of Tregs, IL-10 expression	(131)
BLIMP1	IL-17 expression	Inhibitory histone modifications at IL-17 locus	(111)
CTLA4	It acts as an “off” switch when bound to CD80 or CD86 on antigen-presenting cells, effectively dampening immune responses.	Development and function of naïve Treg cells rather than effector T cells	(135)
IL2RA (CD25)	IL-2/IL-2R pathways, alter susceptibility to autoimmune diseases like multiple sclerosis and rheumatoid arthritis	Affect development and function of naïve Treg cells	(85)
BRG1	Chromatin remodeling factor	Can convert defective Treg cells into powerful, super-activated Treg cells (SuperTreg cells)	(113)
HIP1a	Target FOXP3 degradation	Promote Th17 prevelance over Tregs	(138)
DNMT1	Dnmt1 induces methylation of CNS2 locus	Inhibition of Dnmt1 ensures FOXP3 expression	(139)
TET2	Tet2 inhibits remethylation of CNS2 locus	Tet2 recruited by IL2 to CNS2 prevent remethylation and ensures FOXP3 expression	(139)

Research examines DNA methylation patterns at the FOXP3 locus, a critical epigenetic regulator of Treg development. Methods that inhibit DNA methyltransferases (DNMTs) and promote the activity of ten-eleven translocation (TET) enzymes can increase FOXP3 expression and enhance Treg stability (140). TET enzymes are a group of dioxygenases essential for DNA demethylation. They convert 5-methylcytosine (5mC) into 5-hydroxymethylcytosine (5hmC) through oxidation. This reaction initiates a process that ultimately removes methyl groups from DNA, thereby influencing gene expression and development (141). TET enzymes are crucial for DNA demethylation and play a vital role in establishing Treg-specific epigenetic marks. Manipulating TET enzymes presents a promising strategy for generating and maintaining stable Tregs (142, 143). Modifying histone acetylation and methylation also alters Treg function. For instance, histone deacetylase inhibitors (HDACi) can improve the immunosuppressive capabilities of Tregs (144).

Epigenetic modifications enhance Treg suppressive function, thereby improving immune response regulation in autoimmune diseases and transplantation. Adjusting epigenetic marks should allow the development of Super-Tregs that remain stable over the long term, even amid inflammation. These strategies hold promise for new treatments for autoimmune conditions, transplant rejection, and other immune-related disorders, such as neurodegeneration (131, 145) (**Figures 2**, **3**; **Table 1****).**

### Can MicroRNAs create super Tregs?

miRNAs modulate gene expression post-transcriptionally (146). These small, approximately 22-nucleotide-long, non-coding RNAs are produced in a tissue- and cell-specific manner to regulate nearly all vital functions in metazoan cells by base-pairing with their target mRNAs, leading to the reversible suppression and degradation of these messages (146). Thus, the abundance and activity of miRNAs are crucial for the proper functioning of diverse human cell types, including T cells. Under various conditions, miRNAs undergo significant regulation at both transcriptional and post-transcriptional levels to ensure coordinated control over cell fate and survival (147). In immune cells, the expression of one or more key miRNAs is essential for the development and maintenance of an effective immune system. From managing host-pathogen interactions to guiding the development of specific immune cell subsets, miRNAs play a vital role in ensuring optimal regulation (148). Furthermore, miRNAs from immune cells regulate their and recipient cell functions, influencing immune responses. miRNAs in extracellular vesicles, with anti-inflammatory or immunogenic properties, are valuable for gene regulation in higher eukaryotes (18, 149).

MicroRNAs (miRNAs) play a crucial role in the development and function of regulatory T (Treg) cells, which are essential for maintaining immune balance and preventing autoimmune responses (150, 151). miRNAs regulate gene expression in Tregs, influencing their ability to suppress immune activity—changes in miRNA expression lead to immune system dysfunction (152). In Treg cells, miRNA impairs peripheral homeostasis and suppressor functions, suggesting the importance of miRNA machinery for Treg function (153, 154). Treg development within the thymus or its induction from peripheral T cells requires a functional miRNA network. Among others, miR-181a/b-1, miR-155, and miR-17∼92 have been recognized for their roles in Treg development. miR-181 regulates intrathymic tTreg development (155), miR-155 and miR-17∼92 control Treg-cell homeostasis and function (156). Medullary thymic epithelial cells (mTECs) mature by upregulating MHC class II, CD80, and the autoimmune regulator Aire. This process is essential for central tolerance, as mature mTECs display tissue-specific antigens to developing T cells, promoting the removal of self-reactive T cells (157). Dong et al. suggested that the miR-155–TGFβ axis plays a role in the thymic medulla in determining mTEC maturity and generating Treg cells (158). Therefore, miR-155 ensures proper tTreg cell development. The miR-17∼92 family of miRNAs is crucial for the development of early thymocytes. They promote thymocyte proliferation by inhibiting PTEN translation (159). The miR-17 family also regulates FOXP3 expression, a key transcription factor in Treg development and function. miR-17 can downregulate EOS, a co-regulator of FOXP3, thereby affecting Treg suppressive activity (160) (**Table 2**).

**Table 2:** Key miRNAs in the regulation of Tregs function.

*Candidate miRNA*	Function in Tregs	Level of supporting evidence
***miR-155***	Enhances Treg survival, proliferation, and function by boosting IL-2 signaling through SOCS1 targeting.	*In vitro*, *In vivo* (161–163)(murine)
***miR-146a***	Acts as a negative feedback regulator, crucial for suppressor function by downregulating STAT1, limiting Th1 responses and autoimmunity.	*In vitro*, *In vivo* (164) (165)(murine), Human (166)
***miR-17~92 cluster***	Assists in maintaining Treg fitness and proliferation, though role can be context-dependent and linked to autoimmunity in excess; members target PTEN and CREB1.	*In vitro*, *In vivo* (murine) (160, 167)
***miR-181a***	Functions as an “intrinsic rheostat” for T cell receptor (TCR) signaling, crucial for thymic Treg development and modulating antigen sensitivity.	*In vitro*, *In vivo* (murine) (155, 168, 169)
***miR-31***	A negative regulator of induced Treg (iTreg) differentiation and FOXP3 expression in human cells.	*In vitro*, Human (170, 171)
***miR-142-3p***	Controls GARP expression and secondly influences FOXP3 levels by targeting AC9 mRNA; misregulation can hinder Treg development.	*In vitro*, *In vivo* (murine), Human (172–175)
***miR-10a***	Highly expressed in natural Tregs (nTregs); its expression in iTregs is induced by TGF-β and retinoic acid, which promotes iTreg generation.	*In vitro*, *In vivo* (murine) (176, 177)

Certain miRNAs are mainly expressed in FOXP3+ CD25+ Tregs, with miR-146a being highly present and vital for their immunosuppressive role. Lack of miR-146a in Tregs can impair immune tolerance and promote autoimmune diseases (165). Importantly, miR-146a primarily regulates Tregs through the IFN-γ/signal transducer and activator of transcription (STAT)1 pathway, which plays a role in Treg function to inhibit Th1 responses (164). Without miR-146a, Treg cells lose immunological tolerance, causing severe IFN-γ-dependent immune damage in multiple organs. This is likely due to increased expression and activation of STAT1, a direct target of miR-146a (165). A recent report indicated that miR-146a also upregulates FOXP3 and suppresses inflammation by targeting HIPK3/STAT3, as observed in allergic conjunctivitis (178).

miR-142 plays a vital role as a “checkpoint” miRNA in T regulatory cells (Tregs). Analyzing Tregs lacking miR–142 showed increased expression of several genes involved in the interferon-gamma (IFN-γ) signaling pathway. Multiple genes related to IFNγ were directly targeted by miR-142-3p. In miR-142-3p-deficient Tregs, excessive production and signaling of IFNγ were detected. Removing IFN-γ corrected the Treg homeostasis issues and reduced autoimmunity in FOXP3CremiR-142fl/fl mice. These results suggest that miR-142 is a key regulator of Treg stability, mainly by dampening IFN-γ responses (179). A deficiency in miR-15/16 alters the expression of critical Treg proteins, leading to the accumulation of functionally impaired FOXP3loCD25loCD127hi Treg cells. Without miR-15/16, Tregs proliferate excessively, leading to a shift toward an effector Treg phenotype. Tregs lacking miR-15/16 have difficulty regulating immune activation, which leads to spontaneous multi-organ inflammation and heightened allergic responses in a mouse model of asthma (180). miR-145 in Tregs negatively regulates CTLA-4, key for immune suppression, by binding its transcript’s 3′-UTR in human CD4+ Tregs (181).

Proper regulation of miRNAs in Tregs is key to maintaining immune tolerance and preventing autoimmune diseases (**Table 2**). Understanding their role may lead to new therapies. miRNAs can help generate super-Tregs by targeting genes vital for Treg development (miR-181a), homeostasis (miR-155), and repressive functions (miR-146a) (**Figure 3**).

Super-Tregs should exhibit enhanced miR-146a expression, which strengthens the anti-inflammatory arm of Tregs while reducing NF-κB expression (182), accompanied by a higher IL-10 level (165). Super Tregs, for potent immunosuppressive capabilities, also need to be engineered for improved homing to the brain, and to interact with activated astroglial and microglial cells to modulate the immunosuppressive activities of Th17 and T effector cells. This interaction should lead to decreased cytokine production and less neuronal damage and loss in the brains of patients with Alzheimer’s disease (AD) and Parkinson’s disease (PD). Achieving this will require ectopic expression of certain microRNAs, such as miR-146a and miR-142, or the repression of others, such as miR-145. These approaches may help sustain a balanced expression of FOXP3 and its related genes upstream and downstream, promoting Treg polarization, tissue homing, and enhanced immunosuppressive capabilities in the brain. A revised miRNA profile might enable Tregs to transfer specific miRNAs to nearby glial and neuronal cells via extracellular vesicles or direct cell-to-cell contact via tunneling nanotubes (183), thereby promoting neuroprotection and anti-inflammatory responses in the AD-affected brain. To repress miRNAs detrimental to Treg function, such as miR-24, which directly targets FOXP3 expression (181), employing LNA-modified anti-miRs is a promising approach (184).

CRISPR-Cas9 technology enables the precise alteration of miRNA expression by targeting specific genomic regions, such as the pre-miRNA sequence, to inhibit miRNA production. This approach allows for both knockdown and fine-tuning of miRNA levels, serving as a valuable tool for investigating miRNA functions and exploring potential therapeutic applications (185). CRISPR-Cas9-mediated alteration of miRNA transient expression in their mature forms would enable the transformation of Super Tregs. This should occur before iTregs are either introduced into the affected brain tissue via stereotactic injection or administered intraperitoneally (186). Delivery methods, such as adenoviral delivery or extracellular vesicles, are being investigated to modulate Treg function and possibly treat diseases where Treg imbalance is implicated (187). Adenoviral delivery of miR-21 into Treg cells decreased the expression of both IL-17 and IL-10. A mechanistic study revealed that miR-21 downregulates IL-10 expression by directly targeting it and inhibits the reprogramming of Tregs into IL-17-secreting cells by downregulating STAT3 activity (187). Besides the adenoviral delivery of miRNA expression cassettes to Tregs, one potential alternative for miRNA manipulation is the transient expression of mature miRNAs in Tregs through electroporation (188, 189). A recent development involves a novel electroporation technique that uses a nanostraw electroporation delivery platform, separating the delivery process from biological mechanisms such as endocytosis. This enables the precise regulation of delivered miRNA amounts and demonstrates ratiometric intracellular delivery of miR-181a and miR-27a into primary dermal fibroblasts (188). These strategies can help generate iTregs *ex vivo* from CD25+ FOXP3+ Tregs to obtain super Tregs.

Lipid nanoparticle-based delivery of small single-stranded (ss)RNAs into Tregs could be another strategy for generating super Tregs. In one study, the use of lipid nanoparticles (LNPs) to deliver nucleic acids to the brain releases miR-137 cargo and inhibits the target transcripts of interest in neuroblastoma cells (190). In another study, miRNA mimics were directed to macrophages using LNPs. The encapsulation of miR-146a within a lipid complex protects nucleic acids from nuclease degradation while enabling rapid uptake by target myeloid immune cells. This strategy effectively inhibits the expression of target interleukin-1 receptor-associated kinase 1 (IRAK1) and tumor necrosis factor receptor-associated factor 6 (TRAF6) proteins, leading to decreased NF-κB activity in mouse macrophages *in vitro* (191). Similar results were also observed in rat glioblastoma cells, which inhibited inflammatory cytokines induced by amyloid beta oligomers (192, 193). This strategy may be implemented in Tregs to produce Super Tregs. miR-146a and miR-155 are excellent candidates for repressing immune activation, thereby enhancing Treg activity. An alternative approach involving the knockdown of specific miRNA molecules will equip Tregs with strong anti-inflammatory properties (**Figure 3**; **Table 2****).**

**Figure 4:**
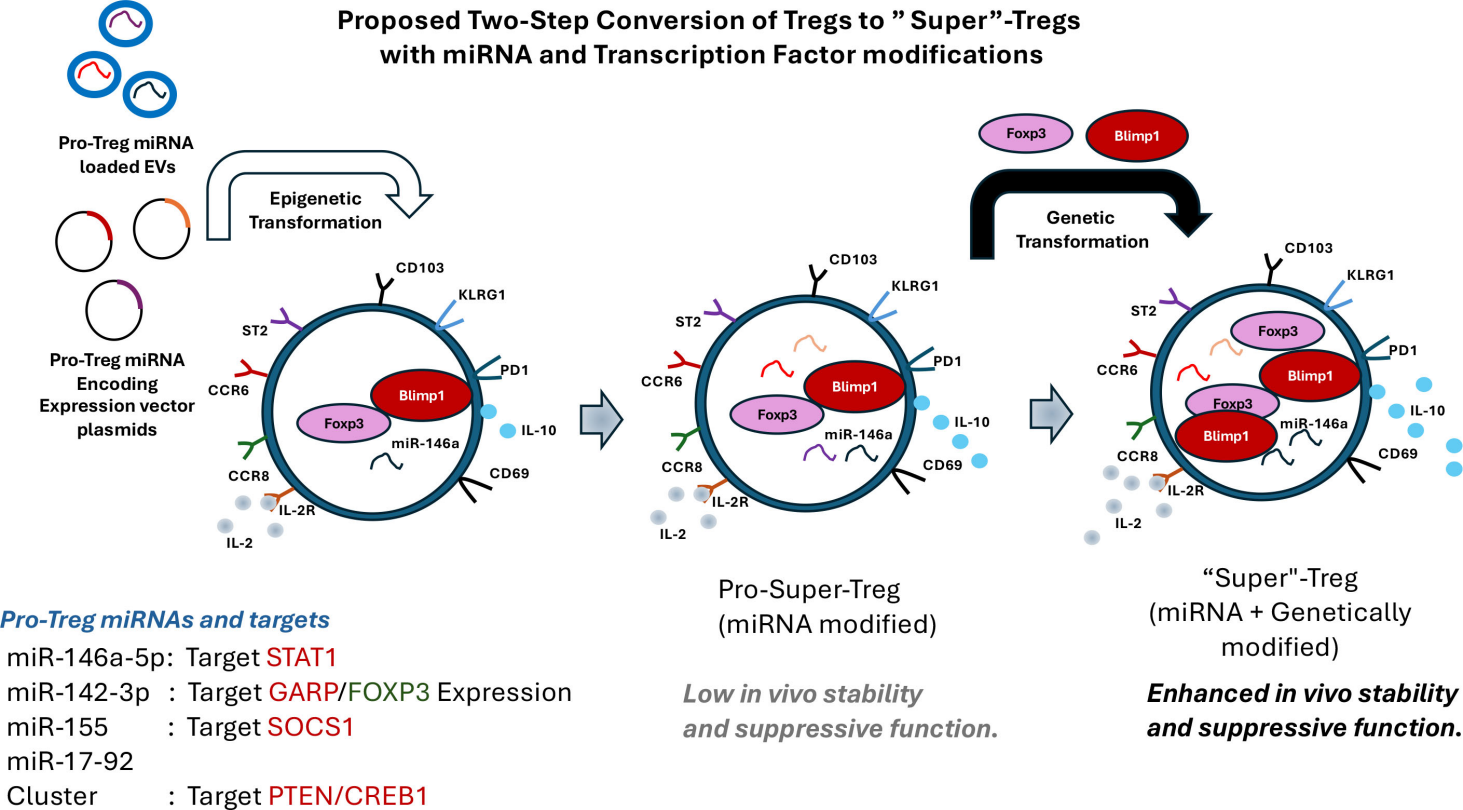
A schematic strategy to transform Tregs into “Super Tregs” with improved stability and suppressive function by a two-step process. Initially, Tregs are converted into Pro-Super Tregs by manipulating cellular miRNA levels; pro-Treg miRNAs condition cells by suppressing target genes that impede Treg function and stability. This can be achieved by introducing an inducible expression vector encoding these miRNAs or by using extracellular vesicles (EVs) that can be delivered to Tregs, regardless of their genetic background, but with altered epigenetic status. In the subsequent step, transcription factors such as FOXP3 or Blimp1 are introduced to modify the transcription profile of the Pro-Super Tregs, transforming them into “Super Tregs” with enhanced survival and anti-inflammatory properties. A list of potential pro-Treg miRNAs and their possible targets in Treg cells is provided.

Delivery or ectopic expression of specific miRNAs to convert Tregs into Super-Tregs may not result in lasting expression of FOXP3 or other Super-Treg markers. Instead, it might only promote Tregs to a pro-state that can later be used to generate Super Tregs via genetic manipulation of transcription factors such as FOXP3 (194). Technically, miRNA expression can be driven by inducible promoters ex vivo in Treg cells, thereby ensuring controlled expression and inducing genetic and epigenetic changes. These changes can suppress critical candidate mRNAs that encode proteins that inhibit the conversion of Tregs to Super-Tregs. Thus, miRNAs can induce Pro-Super-Tregs, which, upon expression of factors like FOXP3, produce “Super” Tregs with improved survival and activity compared to those generated solely by FOXP3 expression. This dual approach of Treg conversion would be an effective strategy for generating “Super”- Tregs (**Figure 4**).

### Extracellular vesicles, miRNA export and super-Tregs

Like many other cells, Tregs shed extracellular vesicles (EVs), and Treg EVs contain a diverse range of cargo, including proteins, lipids, and nucleic acids such as miRNAs (195–198). Treg EVs can transfer molecules to target cells, including effector T cells and dendritic cells, thereby altering their behavior and cytokine production (195–197). The export of miRNAs from cells is crucial for controlling cellular functions. In Tregs, this process may involve two regulatory mechanisms. Exporting particular miRNAs that support Treg survival and their anti-inflammatory role might, in fact, be detrimental to Tregs. Conversely, miRNAs that hinder Treg differentiation and TCR expression could have the opposite impact. Therefore, targeting specific miRNA groups could enable both enhancement and suppression of miRNA activity in Tregs, thereby influencing their differentiation into super Tregs. Tregs release EVs that inhibit immune responses and promote self-tolerance. These Treg-derived EVs transfer molecules, including microRNAs, to target cells, thereby modulating their activity and maintaining immune homeostasis (195–197). Treg-derived EVs are crucial for maintaining immune balance by suppressing excessive immune responses (195–197). Treg EVs have been shown to suppress T-cell proliferation, induce apoptosis, and modulate cytokine expression, ultimately limiting immune responses (195, 196). They contribute to maintaining self-tolerance by transferring molecules to dendritic cells, promoting a tolerogenic phenotype, and reducing inflammation (197). Treg EVs are also being investigated as potential drug delivery vehicles, enabling targeted delivery of therapeutics to specific cells or tissues (199). Despite the promising potential of Treg EVs, several challenges remain, including the need for standardized protocols for isolation and purification, as well as for large-scale production and optimization of the drug-loading efficiency of Treg EVs. Further research is needed to fully understand the mechanisms of action of Treg EVs and their potential clinical applications (196).

Macrophages infected with *Leishmania donovani (Ld)*, a protozoan parasite, were found to exhibit an anti-inflammatory response along with an excess release of miR-146a miRNAs via extracellular vesicles (EVs), which can lead to depolarization of activated macrophages (149). The infected-cell-derived EVs, enriched in miR-146a, were shown to mediate anti-inflammatory responses. Thus, miR-146a-containing EVs derived from macrophages are an excellent source for delivering miRNAs to other immune cells and could serve as a valuable strategy for generating Super-Tregs. The significance of miRNA and non-miRNA factors in anti-inflammatory *Ld*-infected macrophage-released extracellular vesicles (EVs) could be used to create Pro-Super-Tregs (149) (**Figure 3**, **Table 2**).

### Super Tregs function

Tregs inhibit immune responses through various mechanisms, including the secretion of immunosuppressive cytokines like IL-10, TGF-β, and IL-35, which induce cell death via cytolysis, deplete resources to alter the metabolic environment, and modify the activity of antigen-presenting cells (APCs), such as dendritic cells (26, 35, 200). Tregs produce IL-10, TGF-β, and IL-35, which inhibit the activation and proliferation of other immune cells, effectively reducing the overall immune response (201). Tregs can also kill target cells, including other T cells, using granzymes and perforin, much like cytotoxic T cells eliminate infected or cancerous cells (35, 202). Interestingly, Tregs also compete with other immune cells for glucose and amino acids, effectively starving them and hindering their activation and proliferation. Additionally, Tregs can produce adenosine, an immunosuppressive molecule that inhibits the activation and function of other immune cells (203). Tregs can interact with dendritic cells (DCs), which are crucial for initiating immune responses. This interaction leads to the suppression of DC maturation and activity, thereby reducing the chances of activating an immune response. The surface molecules CTLA-4 and LAG-3 present on Tregs engage with DCs, potentially modulating their function and preventing their excessive activation (204, 205). Tregs express the high-affinity IL-2 receptor (CD25), which enables them to bind and sequester IL-2, a cytokine essential for the survival and activation of other T cells (206).

### Limitations of super Tregs-based therapy

Super Tregs, which are exceptionally potent regulatory T cells, present various challenges for clinical use. These include A. maintaining stability and functionality *in vivo*, B. avoiding possible off-target effects, and C. effectively producing and administering them. Moreover, D. the plasticity and instability of Tregs, E. their potential for inducing immune responses, and F. the necessity for ex vivo expansion create obstacles to their broader application (105). Treg therapy is limited by the scarcity of Treg cells, which constitute only 5% of circulating CD4 T cells. Generating therapeutic doses needs extensive *ex vivo* expansion. Protocols typically involve broad TCR stimulation in magnetically separated or flow-sorted Tregs, with IL-2 and rapamycin, for up to 36 days (207).

In inflammatory environments, Tregs can transform into Th-like Tregs, reducing their suppressive function and potentially increasing inflammation. The epigenetic landscape of Tregs can also change under such conditions, affecting their stability and function (208). The forkhead box protein P3 (FOXP3) is a crucial regulator of Treg function, and its expression can become unstable, leading to a loss of suppressive function. Super-Tregs can suppress beneficial immune responses or worsen tissue damage if not carefully engineered or administered. Producing sufficient Tregs for therapeutic use requires cell expansion, which can potentially compromise their suppressive functions (209). Genetic modifications in Super-Tregs can trigger an immune response, leading the body to attack the altered cells or gene transfer vectors. Additionally, the presence of non-Treg cells during expansion may cause off-target effects, reducing the therapy’s efficacy. Furthermore, large-scale production, transport, and administration of these cell therapies present significant logistical challenges (208). Excessively suppressive Tregs can hinder pathogen clearance and heighten susceptibility to infections (210). Super-Treg effectiveness may vary depending on the specific disease and the patient’s immune status (211). Super-Tregs can be harmful to certain tissues if they are not accurately targeted or if they lose their suppressive function (208). Inflammatory mediators can impair Treg function and stability (212), thereby compromising Treg suppression and potentially exacerbating the inflammatory response. Tregs can become unstable and lose their suppressive function due to chronic inflammation (213).

### Implication for the gut-brain axis

The gut-brain axis interacts with Treg cells, influencing immune responses and overall health. The gut microbiota is essential for promoting the development and function of Treg cells, which support the equilibrium of immune responses in both the gut and the brain. This connection is crucial for maintaining homeostasis but can be disrupted in various diseases (214–216). The gut-brain axis refers to the two-way communication between the gut and the central nervous system (217). This communication occurs through multiple pathways, including the nervous, immune, and neuroendocrine systems (218). The gut microbiota, the community of microorganisms residing in the gut, plays a crucial role in modulating this communication (217). Tregs are crucial to the gut, playing a vital role in maintaining tolerance to commensal bacteria and preventing excessive immune reactions to food antigens. Furthermore, Treg cells can migrate to various tissues, including the brain, where they assist in regulating immune responses (215, 219–223). The gut microbiota plays a vital role in shaping the development and function of Treg cells. Certain gut bacteria can boost the production of Tregs in the gut, enabling these cells to migrate to the brain and influence immune responses. Disturbances in the gut microbiota, whether caused by antibiotics or an unhealthy diet, may change Treg cell populations and disrupt immune balance, potentially impacting both gut and brain health (214, 215, 224). Gut microbiota promotes distal tissue regeneration via RORγ+ regulatory T cells. The microbiome-dependent regulation of Tregs and Th17 cells in the mucosa has been observed in mice (224). A previous report showed that the proinflammatory genus Collinsella, a risk factor for Alzheimer’s, was positively linked to the APOE rs429358 risk allele, confirming genetic ties between Alzheimer’s and gut microbiome genera (225). Understanding the gut-brain axis and Treg cells is vital to the study of diseases such as inflammatory bowel disease, neuropsychiatric conditions, and neurodegenerative disorders. Interventions targeting the gut microbiota, such as probiotics or immunotherapy, may affect the effectiveness of Treg cells. Research shows certain probiotics can reduce depression and anxiety symptoms by affecting gut microbiota and Treg cells (226–228).

The interaction between T cells in the gut lining and the gut microbiota is a potential source for generating and modifying Tregs. Consequently, exposing these T cells to EVs from natural sources enriched in specific proteins or miRNA components that modulate miRNA function in Tregs may offer a promising approach for transforming gut-associated Tregs into Super Tregs. This approach provides a natural means of cultivating Super Tregs through dietary modifications. Milk EVs play a significant role in combating gut inflammation (229), making them compelling candidates for oral gavage administration to achieve Treg genesis in the gut lining.

### Tregs homing in on the degenerating brain: can super Tregs be advantageous?

The tissue homing of Tregs to peripheral tissue niches involves interactions with other immune and non-immune cells (230). The Treg homing process is crucial for their localized actions, which include suppressing inflammation, promoting tissue repair, and preventing autoimmunity (231–233).

How do Tregs control the homing process? IL-18/IL-18R signaling is critical for the induction of the key thymus-homing chemokine receptor CCR6 expression on Tregs and its retention in the thymus (234). Tregs express various chemokine receptors across tissues to help them remain or migrate effectively. For example, the lymph node-homing receptor CCR7 and the integrin alphaL are essential for guiding regulatory T cells into lymph nodes. Additionally, the β2-integrin lymphocyte function-associated antigen-1 (LFA-1) is vital for T cell-antigen-presenting cell interactions. It also facilitates T-cell adhesion to the endothelium, a key step in lymphocyte exit from the bloodstream and their entry into target tissues (235, 236). Tregs also naturally “anchor” themselves in tissues by interacting with components of the extracellular matrix. Layilin, which is a C-type lectin-like receptor, is present on a special group of activated Tregs found in the skin. It plays a vital role in helping Tregs attach firmly to the extracellular matrix with the help of LFA-1 (237).

Brain-resident Tregs express CX3CR1 and CXCR3, which are tissue-homing receptors (238). Tregs are found at the brain’s border areas, such as the meninges, where they function as guardians by regulating immune responses and protecting the brain from injury and infection. Recent findings reveal that the meninges contain a distinct subset of regulatory T cells that help maintain the brain’s microenvironment stability. These meningeal Tregs are highly heterogeneous in their gene expression, yet they play a vital role in limiting the production of IFN-γ by nearby lymphocytes. When Tregs are temporarily removed, immune cells can enter the brain tissue, triggering activation of supportive glial cells and causing damage through IFN-γ–mediated death of nerve-building cells in the hippocampus, which may also affect short-term memory (223). Tregs also interact with tissue-specific cells and release mediators, which contribute to tissue protection (239). CXCR3 is a crucial chemokine receptor regulating the homing of Treg cells. It has been demonstrated that CXCR3 expression in regulatory T cells facilitates interactions with type I dendritic cells in tumors, thereby restricting CD8^+^ T cell antitumor immunity (240). Another report documented the crucial role of CXCL14 in promoting regulatory Treg-cell activation in stroke and its abundance in the affected brain region, where it is regulated via the IL-2/IL-2R pathway (241). Brain astrocytes are specialized cells that maintain high FOXP3 expression in Treg cells through IL-2/STAT5 signaling. This shows how astrocytes and Treg cells collaborate to maintain Treg cell identity in the brain, underscoring their essential interplay (242).

Super-Tregs with enhanced immunosuppressive activity should have better brain-homing ability. Improving CXCR3, CXCL14, or IL-2R may enhance Treg cell brain targeting. Using miRNAs and EVs to modulate Tregs could effectively enhance their brain homing. miR-34a-5p directly affects CXCR3 in T cells and macrophages (243). Downregulating miR-34a-5p boosts CXCR3 in Super Tregs, improving their brain-homing ability.

### Challenges ahead and future of super-Tregs in neurodegeneration

Treg therapies face challenges like generating enough Tregs, targeting the brain accurately, and replacing resident Tregs in time. Their antigen specificity for proteins like amyloid or Lewy bodies can be problematic. Ensuring Tregs have strong immunosuppressive activity, tuned for effective microglia, Tregs, and Teffs interactions, is crucial. Engineering TCR-Tregs to target Aβ or Lewy bodies can reduce amyloid and off-target effects. Tregs should help clear plaques and activate microglia without causing excessive inflammation.Meanwhile, they should ensure that neurons are protected from deposited amyloid protein or inflammatory cytokines. Tregs should only partially restrict microgliosis and astrogliosis, as their powerful function and abundance in the AD brain may have a long-term impact on immunosuppressive activity, possibly delaying the removal of damaged cells crucial for neuronal function, which could also lead to brain tumor development. Recent reports indicate that high glucose levels impair cognitive function by inducing mitochondrial calcium overload in Tregs (244), suggesting a direct link between Treg activity, cognitive brain function, and the metabolic status of immune cells.

Designing effective cell-based neuroprotective therapy with optimal immunogenic properties is challenging. Choosing the right antigen target and creating a cell that recognizes it without off-target effects is crucial. Ensuring Tregs keep their regulatory and suppressive functions *in vivo* is essential. Delivering Tregs to the brain safely is another challenge, as the blood-brain barrier limits their access in AD and PD models. Long-term safety and efficacy must be evaluated before clinical trials (245–247).

Mitigation strategies for challenges in regulatory T cell (Treg)-based therapy focus on improving stability, specificity, and persistence through advanced bioengineering and combination therapies. Functional instability or plasticity can be addressed by genetically modifying Tregs to resist inflammatory signals or by overexpressing stabilizing transcription factors such as FOXP3 and HELIOS, thereby preventing their conversion into pro-inflammatory effector cells (248). Specific cell culture conditions, such as the presence of rapamycin, help maintain a stable Treg phenotype during *ex vivo* expansion (207). Poor persistence or migration is another challenge that must be managed before therapy deployment, which could be achieved, for example, by administering low-dose interleukin-2 (IL-2) or engineered IL-2 muteins to promote Treg survival and expansion *in vivo* without significantly stimulating effector T cells (249). Bioengineering methods, such as “nanogel backpacks” that release IL-2 upon activation, can also enhance persistence and local function (250). Obstacles related to manufacturing and supply logistics can be addressed by implementing robust protocols, including advanced cryopreservation techniques, to ensure cell viability and enable on-demand “off-the-shelf” allogeneic products that bypass some autologous supply chain issues. Combining Treg therapy with immune strategies or innovative engineering, like synthetic Notch receptors, may overcome resistance from certain effector T cells that challenge suppression. Evidence shows Treg therapy works best when combined with other methods for long-term tolerance. Emerging approaches include antibody-based therapeutics (e.g., IL-2 muteins or anti-TNFRSF25 antibodies) to expand a patient’s endogenous Tregs, avoiding complex *ex vivo* manipulation manufacturing (251).

## Conclusion and future research directions

Tregs are crucial in counteracting neuroinflammation and autoimmunity, with Super-Tregs being the most effective for treating AD and PD. The balance between T effector cells (Teffs) and regulatory T cells (Tregs) is vital for managing inflammatory processes that clear amyloid deposits and protect neurons. Early Treg-cell intervention with Super Tregs can reduce inflammatory cytokines in AD or PD brains, thereby protecting neurons. Teffs also activate microglia to remove amyloid proteins. Understanding Teffs, Tregs, microglia, and neurons interactions is key to reversing AD and PD symptoms through Super Tregs.

Using miRNA-mediated Treg transformation into SuperTregs and EVs as miRNA carriers shows promise, but challenges remain. Efficient delivery of EVs to tissue Tregs, particularly across the BBB and to target cells, remains challenging. Engineering EVs with surface proteins for tissue homing and Treg-cell targeting, along with methods such as focused ultrasound (FUS), can facilitate EV entry into the brain (252). Minimizing off-target effects of miRNAs delivered via EVs is crucial to achieving clinical outcomes such as reduced neuroinflammation and cytokine repression by Super-Tregs. The same FUS strategy could be applied to *ex vivo* engineered Super-Tregs to improve homing and control inflammation in AD and PD brains.
